# The use of patient sitters at a Swiss hospital: A retrospective observational study

**DOI:** 10.1371/journal.pone.0287317

**Published:** 2023-06-14

**Authors:** Iris Kramer, Maria Schubert

**Affiliations:** Institute of Nursing, School of Health Sciences, ZHAW Zurich University of Applied Sciences, Winterthur, Switzerland; University of Verona, ITALY

## Abstract

**Objective:**

Patient sitters are frequently used in acute care hospitals to provide one-to-one care for agitated or disorientated patients to assure the safety and well-being of patients. However, there is still a lack of evidence on the use of patient sitters, especially in Switzerland. Therefore, the aim of this study was to describe and explore the use of patient sitters in a Swiss acute care hospital.

**Methods:**

In this retrospective, observational study we included all inpatients who were hospitalized between January and December 2018 in a Swiss acute care hospital and required a paid or volunteer patient sitter. Descriptive statistics were used to describe the extent of patient sitter use, patient characteristics, and organizational factors. For the subgroup analysis between internal medicine and surgical patients Mann-Whitney U tests and chi-square tests were used.

**Results:**

Of the total of 27’855 included inpatients, 631 (2.3%) needed a patient sitter. Of these, 37.5% had a volunteer patient sitter. The median patient sitter duration per patient per stay was 18.0 hours (IQR = 8.4–41.0h). The median age was 78 years (IQR = 65.0–86.0); 76.2% of patients were over the age of 64. Delirium was diagnosed in 41% of patients, and 15% had dementia. Most of the patients showed signs of disorientation (87.3%), inappropriate behavior (84.6%), and risk of falling (86.6%). Patient sitter uses varied during the year and between surgical and internal medicine units.

**Conclusions:**

These results add to the limited body of evidence concerning patient sitter use in hospitals, supporting previous findings related to patient sitter use for delirious or geriatric patients. New findings include the subgroup analysis of internal medicine and surgical patients, as well as analysis of patient sitter use distribution throughout the year. These findings may contribute to the development of guidelines and policies regarding patient sitter use.

## Introduction

Patients in acute care hospitals who are disoriented or agitated often require increased care and observation and/or restraints to ensure their safety and well-being and to prevent adverse or harmful events for themselves or others, due to their dangerous or inadequate behavior [1–3]. In the past, physical restraints such as belt fixations of extremities or bed rails have been used as a first choice intervention for patients with agitated or potentially dangerous behavior [4, 5]. However, from an ethical point of view, this approach is highly controversial [4]. A growing body of evidence also suggests that the use of restraints increases rather than decreases the risk of harm for patients by, for example, increasing the risk of falls or injuries or even death [6, 7]. In these situations, constant, continuous, or close one-to-one observation of the patient provided by so called patient sitters has become a common alternative to the use of physical restraints [8]. In the evidence, there is no unified term, widely accepted definition or concept available for this kind of observation [9, 10]. Commonly used terms are “patient sitter” or simply “sitter,” “specialling” or “specials,” or constant, continuous, close, or one-to-one observation [2, 11]. For this study the term “patient sitter” is used as it is most similar to the German term “Sitzwache,” of which the literal translation would be “sitting guard.”

In Switzerland, where this study was conducted, patient sitters are usually nursing assistants, nurse aides, healthcare assistants, or medical students, sometimes registered nurses (RNs) [2]. This type of one-to-one care may also be provided by persons who are volunteers or family members [12, 13]. Many hospitals use multiple types of patient sitters [14, 15]. The role and activities of a patient sitter can vary a great deal depending on vocational training, skill level, and institutional policies. There are significant differences in the training of patient sitters [12, 13, 15]. The primary responsibility of a patient sitter is observing the patient in order to prevent falls, medical equipment removal, exit seeking and psychiatric crisis, but this role can be more passive or more active [11]. While some patient sitters provide all levels of nursing care, including assistance with activities of daily living and providing diversional and therapeutic activities, others may simply “sit” with the patient and call the assigned nurse whenever the patient requires care [3, 11].

In the evidence, internationally, the duration of patient sitter use varies from one hour [16] up to 120 days per patient [11], and from 1,152 to 24,890 overall hours in general hospital settings [15].

Patients typically require a patient sitter due to significant cognitive impairment, challenging behavior, or some kind of risk, such as risk of falls, risk of self-harm or suicide, risk to others, or elopement [17]. The most common reasons for patient sitter use in acute care are patients with dementia and delirium [2]. Patients with head injuries, neurological problems, or confusion are, according to Wood et al. [2] the second most common category. Other groups who require the use of patient sitters include patients with substance use disorders, patients undergoing alcohol withdrawal, or patients experiencing some sort of mental distress [2]. Challenging behavior that may require a patient sitter includes patient agitation, pulling on tubes or other medical devices, and confusion or disorientation [1]. Solimine et al. [1] found different reasons for patient sitter use depending on the age of the patient. Whereas youths and adults aged under the age of 65 were most likely to require patient sitters due to suicide risk, elderly patients (> 65 years) were most likely to need a patient sitter due to risk of falls and delirium [1]. Another established, though less frequently recognized, role for (often volunteer) patient sitters, is the provision of companionship for patients in palliative or terminal care [18].

Beside the patient characteristics and behaviors, nurse staffing seems to be a relevant influencing factor on patient sitter use as well [19, 20]. High rates of RN overtime, as well as RN and nursing assistant understaffing were associated with greater use of patient sitters [20]. In another study, higher level of experience among RNs was associated with a reduction in patient sitter use [19].

Although the use of patient sitters seems to be a common practice in hospitals nationally and internationally, the evidence on the use of patient sitters is still limited. Therefore, the aim of this study was to describe and explore the use of patient sitters in a Swiss acute care hospital in general and in the subgroups of surgical and internal medicine patients based on socio-demographic and clinical factors and to compare the surgical and internal medicine departments based on organizational factors contributing to patient sitter use. Thus, this study provided important insights into the use of patient sitters at a Swiss acute care hospital and into group- and season-related differences of patient sitter use.

## Materials and methods

In order to achieve the study aim, we conducted a quantitative, retrospective observational study with a descriptive approach using routine patient data.

### Study setting

The study was carried out in a regional, cantonal acute care hospital in Switzerland with 500 beds and approximately 28,000 inpatients per year. The hospital comprises the following departments: internal medicine; surgery; obstetrics and gynecology; children and youth; and various institutes (eye clinic, radiation oncology, and emergency). The internal medicine and surgical departments treat most patients and have the highest use of patient sitters. As such, these departments were of particular interest for this study.

### Study sample

All inpatients who required a paid or volunteer patient sitter for at least four hours at the study hospital between January 1, 2018 and December 31, 2018 were included in the study. The minimum of four hours for patient sitter use was defined because it mainly considered longer periods of patient sitter use where the patient sitters were not spontaneously assigned but rather planned and thus required extra resources. Patients in the following units were excluded: intensive care; intermediate care; post-anesthesia care; neonatal intensive care; and delivery room. These units typically have a higher patient–nurse ratio, and very often a one-to-one ratio, where it is not considered a patient sitter use.

### Patient sitters in the study hospital

Typically, paid patient sitters in the study hospital are individuals who have successfully completed or are in the process of completing a nursing internship (often medical or nursing students), healthcare assistants, and nursing assistants. Their internship or training qualifies them to be patient sitters without further education. They are managed in a pool and sometimes requested using an in-house text message list; alternatively, paid patient sitters are made available by nurse managers of other nursing units. In addition, the chaplaincy of the hospital manages a team of volunteer patient sitters who exclusively cover night shifts, except for the palliative care unit, where they also provide short-term daytime patient sitter use. The volunteer patient sitters complete a one-week course focusing on spiritual care and some aspects of end-of-life care. They do not receive any payment and are not family members of the patients. These volunteer patient sitters can be requested on a daily basis every morning. Decisions to order a patient sitter (volunteer or paid) are therefore made daily and apply to a specific shift or time period, sometimes on very short notice. RNs play a key role in making decisions related to ordering a patient sitter. A medical order is not necessary, and no written policy on patient sitter use is available. No guidance exists concerning the characteristics of patients for whom a patient sitter should be used in general, the type of patient sitter (volunteer or paid) required, or the role and responsibilities of a patient sitter. However, in an unofficial understanding, the patient sitters fulfil the known tasks of increasing patient safety by making sure that patients do not stand up on their own and fall unobserved, that they do not injure themselves or remove tubes and generally ensure the patients well-being. Further, the responsibilities and tasks of a patient sitter may vary based on the sitter’s vocational training, resulting in a more active role for paid patient sitters and a more passive role for the volunteer patient sitters. A patient may receive one-to-one care from only paid, only volunteer or both, paid and volunteer, patient sitters during his or her hospital stay depending on the level of care that is needed and the current availability of patient sitters.

### Study variables

The following main outcome variables were selected to describe the extent and usage of patient sitters: total number of patients who had a patient sitter; duration of patient sitter use per patient per stay; and number of patient sitter uses per patient per stay. All these variables were grouped into paid, volunteer, and both paid and volunteer patient sitters. The use of a patient sitter was not documented as a standard, hence the reason for the need of a patient sitter was not documented, either. Therefore, we could not include the reason for a patient sitter as such as a study variable and instead selected patient characteristics and other descriptive variables as described hereafter. To describe the characteristics of patients who had a patient sitter, we included the sociodemographic variables age and sex, as well as the following clinical variables: medical discipline; unit; main medical diagnosis, according to the code chapters of the tenth version of the International Classification of Diseases, German modification (ICD-10-GM [21]); and medical diagnoses that typically increase likelihood of patient sitter use (dementia, delirium, disorders due to use of alcohol, agitation and violence, and suicidal ideation). To explore the relevance of patient sitters in end-of life and terminal care, the variable in-hospital mortality was included. Other patient descriptor variables consisted of variables describing patient behavior and risk factors which indicate use of a patient sitter, such as disorientation (patient shows signs of verbal or behavioral manifestations of not being oriented to time or place or of misperceiving persons in the environment); inappropriate behavior (patient displays symptoms of inappropriate behavior toward the place and/or the person, e.g., pulling at tubes or dressings or attempting to get out of bed when doing so is contraindicated); limited orientation (patient shows signs of not being oriented to all three dimensions of orientation, i.e., time, place, and person); risk of falls; and intake of psychotropic drugs (drugs that increase risk for falls or delirium). Other study variables of interest were organizational factors that may contribute to patient sitter use, such as nursing workload (the sum of direct nursing care per month per unit in minutes–an automatically calculated number of the time used for all nursing tasks documented by nurses) and the number of patient sitter uses per month per unit, grouped into volunteer and paid patient sitters.

### Data collection and data sources

All patient data was recorded on a routine basis in the hospital information system by health professionals and administration staff. Authorized personnel extracted the required data from the following five databases which are part of the hospital information system, and provided them encrypted to the research team: PRISMA database (Patient Record in Somatics), the Swiss Federal Statistical Office medical and administrative database [22]; the LEP database (“Leistungserfassung in der Pflege” / nursing performance classification), which is used for time keeping documentation of nursing activities related to patient care by nurses; the ePA-AC database (“ergebnisorientiertes PflegeAssessment acute care”/ outcome-oriented nursing assessment in acute care), a research-based standardized method for assessing and displaying a patient’s impairments and abilities through points and scores by nurses [23]; the Nu-DESC database (Nursing Delirium Screening Scale, German version [24]), an observational five-item assessment instrument used to screen for delirium; and the chaplaincy record of all volunteer patient sitter uses. The PRISMA database provided data about age, sex, medical discipline, and medical diagnosis. Data about the extent and usage of paid patient sitters and organizational factors including nursing workload was provided in the LEP database. The hours of paid patient sitter use were determined by the LEP-variable "1:1-care" which provided the number of minutes of paid patient sitter use per shift per patient documented by nurses. The number of hours of volunteer patient sitter use were extracted from the chaplaincy record. The ePA-AC database comprised the study variables limited orientation, risk of falls, and intake of psychotropic drugs. The Nu-DESC database was used for the study variables disorientation and inappropriate behavior. The chaplaincy record provided data about the extent and usage of the volunteer patient sitters. Because patient sitter use is not documented in a standard manner, eligible patients were identified by filtering the LEP database using the variable “1:1-care,” though this only identified paid patient sitters. Therefore, all patients from the chaplaincy record were used as a filter for the other databases, too. The data sets were linked by case identification numbers or by unit for organizational factors.

### Data analysis

Descriptive statistics were used to determine the extent and usage of patient sitters, the sociodemographic and clinical characteristics of patients who needed a patient sitter, and organizational factors. Because all variables were non-normally distributed, we used medians (med) with interquartile ranges (IQR) for continuous variables. Frequencies and percentages were stated for categorical variables.

To test for statistically significant differences between internal medicine and surgical patients who required a patient sitter, the non-parametric Mann-Whitney U test was used for continuous variables. This test was used due to non-normally distributed data according to the Kolmogorov-Smirnov test [25]. Nominal variables were tested with chi-square independence tests. Yates’ correction was used for all 2 x 2 tables; where more than 20% of all cells had expected frequencies < 5, Fisher’s exact test was used [26]. The two-sided level of significance for all tests was set at alpha < .05. Because of the exploratory nature of the subgroup analysis, we forwent multiplicity adjustment [27]. Data analyses were carried out using the statistical software program IBM SPSS Statistics (Statistical Package for Social Sciences) version 26 [28].

### Ethical considerations

This study was conducted in compliance with the Declaration of Helsinki [29], the Guidelines for Good Clinical Practice (GCP) by the International Council for Harmonisation of Technical Requirements for Pharmaceuticals for Human Use [30], and all national legal and regulatory requirements, such as the Federal Act on Data Protection. This study was part of a multi-center health service research program on restraints. It was reviewed by the Ethical Committee of the Canton of Zurich (BASEC-nr. Req-2019-0030). The study was classified as not falling under the Swiss Human Research Act because only encrypted, routine patient data, collected on a regularly basis, were used. According to national guidelines, no authorization from the Ethical Committee is required for such a project. Furthermore, according to national and institutional guidelines for the use of these data, no informed consent is required.

## Results

### Extent of patient sitter use

**Fig 1:**
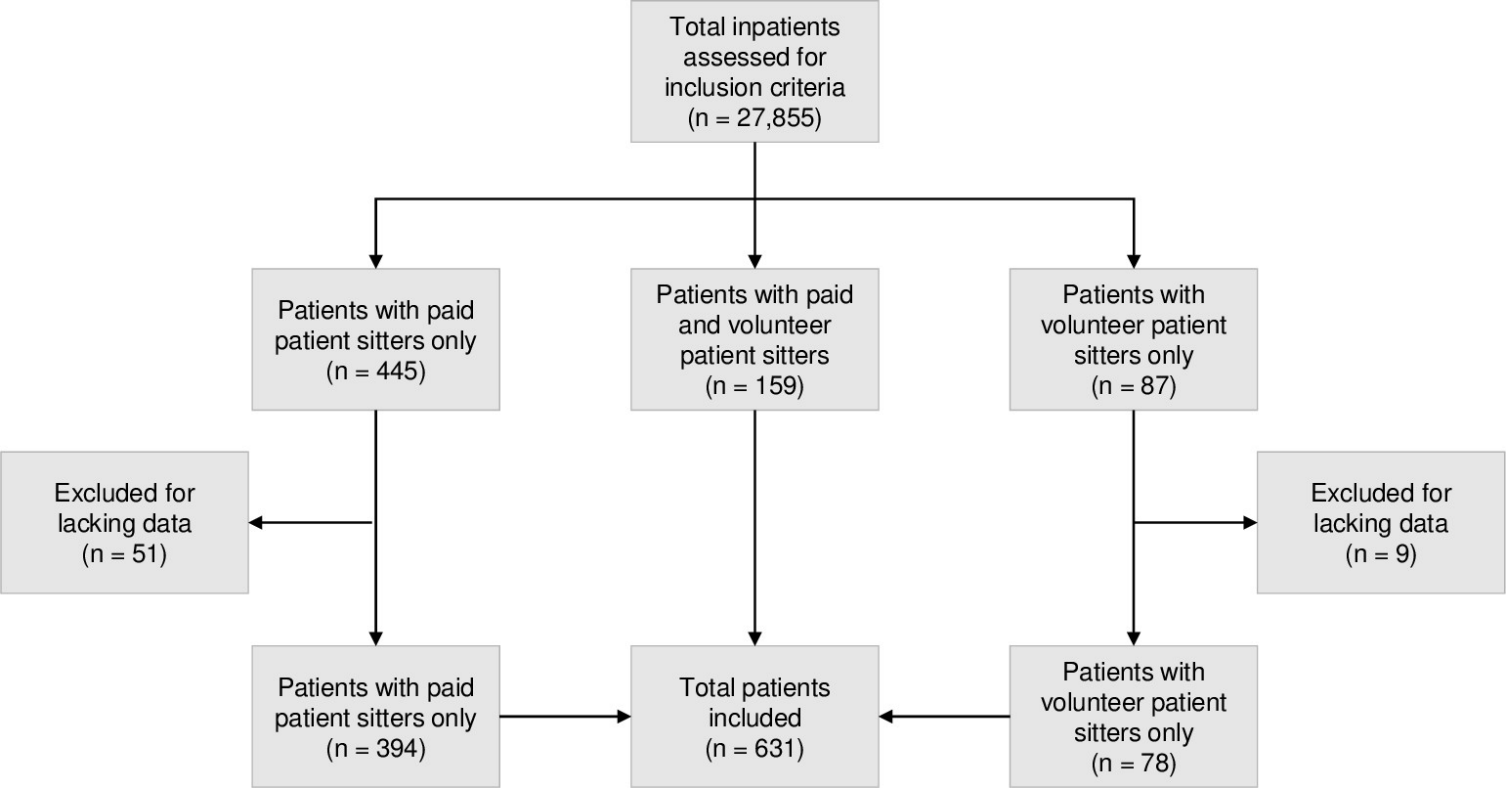
Study flow chart. The study flow chart shows the total number of inpatients whose eligibility were assessed against the inclusion criteria; divided into patients who had both paid and volunteer patient sitters (center), and those who had either a paid (left) or a volunteer (right) patient sitter. The bottom center shows the total number of patients included in the study.

A patient sitter was used for 2.3% of the 27,855 included inpatients in the study period, as shown in Fig 1, which displays the extent of patient sitters used in the study hospital. Of the patients who had a patient sitter, 87.6% had a paid patient sitter and 37.5% had a volunteer patient sitter. 25.2% patients had both, paid and volunteer patient sitters. A total of 60 (8.6%) patients with patient sitters had to be excluded from the study due to missing data in one of the databases. The median duration of patient sitter use per patient per stay was 18.0 hours (IQR = 8.4–41.0 hours) with a maximum outlier of 669.6 hours (27.9 days) and a minimum of 4 hours. The median number of patient sitter uses per patient per stay was 2.0 (IQR = 1.0–5.0; min = 1.0; max = 50.0).

### Characteristics of patients with patient sitter use

The characteristics of patients with a patient sitter (paid and volunteer) are shown in Table 1. Median age was 78 years, and more than three-quarters of patients were over the age of 64. Gender distribution was almost equal. Most patients who required a patient sitter were in the internal medicine or surgical department, and of the included units the trauma unit had the most patient sitter uses (n = 70; 12.5%). The top three main medical diagnoses fell within the ICD-10 chapters “injury, poisoning of external cause,” “neoplasms,” and “diseases of the circulatory system.” Of the selected diagnoses that typically increase likelihood of patient sitter use, delirium was the most common, followed by dementia and disorders due to use of alcohol. According to the nursing assessments, more than 85% of patients requiring a patient sitter showed signs of disorientation, inappropriate behavior, and risk of falls. Suicidal ideation was diagnosed in 1.3% of patients with a patient sitter, whereas the rate of patients with suicidal ideation in all inpatients was 0.2% (n = 57/27,855). In-hospital mortality rate was 11.8% for patients with a patient sitter, and the overall in-hospital mortality rate for all inpatients was 2%. The in-hospital mortality rate was higher for patients with a volunteer patient sitter than for patients with a paid patient sitter.

**Table 1:** Patient characteristics.

	N = 631
**Age** (years), *med* [IQR]	78.0 [65.0–86.0]
< 18 years, *n*(%)	38 (6.0)
18–64 years	112 (17.7)
> 64 years	481 (76.2)
**Sex, *n*(%)**	
Female	311 (49.3)
Male	320 (50.7)
**Medical discipline**, *n*(%)	
Internal medicine	288 (45.6)
Surgery	267 (42.3)
Children and youth medicine	35 (5.5)
Eye clinic	21 (3.3)
Obstetrics and gynecology	14 (2.2)
Radio oncology	6 (1.0)
**Main medical diagnosis ICD-10 chapters**, *n*(%)	*n* = 620
Injury/poisoning of external causes	139 (22.4)
Neoplasms	109 (17.6)
Diseases of the circulatory system	75 (12.1)
Mental and behavioral diseases	49 (7.9)
Diseases of the respiratory system	40 (6.5)
Diseases of the musculoskeletal system	38 (6.1)
Diseases of the nervous system	30 (4.8)
Diseases of the genitourinary system	29 (4.7)
Diseases of the digestive system	25 (4.0)
Diseases of the eye and ear	19 (3.1)
Other ICD-10 chapters	67 (10.6)
**Medical diagnoses** *(multiple diagnoses per patient possible)*, *n*(%)	
Delirium	259 (41.0)
Dementia	95 (15.1)
Disorders due to use of alcohol	47 (7.4)
Agitation, physical violence	8 (1.3)
Suicidal ideation	8 (1.3)
**Behaviors and risk factors for patient sitter use**, *n*(%)	
Disorientation (*n* = 487)	425 (87.3)
Risk of falls (*n* = 514)	445 (86.6)
Inappropriate behavior (*n* = 487)	412 (84.6)
Limited orientation (*n* = 509)	370 (72.7)
Intake of psychotropic drugs (*n* = 513)	154 (30.0)
**In-hospital mortality**, *n*(%)	
Patients with patient sitter (*n* = 620)	73 (11.8)
Patients with volunteer patient sitter (*n* = 234)	36 (15.2)
Patients with paid patient sitter (*n* = 542)	62 (11.2)
All inpatients (*n* = 27,553)	556 (2.0)

*Note*. *n* = sample size; *med* = median; IQR = interquartile range

### Differences between surgical and internal medicine patients

The differences between the subgroups of surgical and internal medicine patients requiring a patient sitter are shown in Table 2. The duration of paid patient sitter use was significantly higher for internal medicine patients than for surgical patients. There was no statistically significant difference for other types of patient sitters in duration or number of patient sitter uses per patient. Surgical patients tended to be significantly older than internal medicine patients. We also found a statistically significant difference between the diagnoses dementia and delirium and the medical discipline of the patients requiring a patient sitter. Dementia was more common in surgical patients than internal medicine patients, while delirium was more common in internal medicine patients than surgical patients. The two subgroups differed statistically significant by their main medical diagnosis. While the most frequent diagnoses related to the ICD-10 chapters for surgical patients were injury/poisoning of external cause, neoplasms, and diseases of the circulatory and musculoskeletal system, the most frequent diagnoses for internal medicine patients were neoplasms, diseases of the circulatory system, and mental and behavioral disorders. In-hospital mortality was significantly higher for internal medicine patients requiring a patient sitter than for surgical patients.

**Table 2:** Differences between surgical and internal medicine patients with patient sitter.

		Surgical patients *N* = 267	Internal medicine patients *N* = 288	Test statistics	*p* value
**Duration of patient sitter use** (hours), *med* [IQR]					
Paid and volunteer patient sitters		40.3 [26.4–61.3]	48.4 [27.8–82.6]	Z = -1.737	.082
Paid patient sitter		22.5 [11.9–43.3]	23.7 [8.7–50.0]	Z = -2.590	**.01**
Volunteer patient sitter		9.0 [9.0–18.0]	18.0 [9.0–36.0]	Z = -.927	.354
**Number of patient sitter uses per patient**, *med* [IQR]					
Paid and volunteer patient sitters		5.0 [3.0–6.3]	5.0 [3.3–8.8]	Z = -1.068	.285
Paid patient sitter		2.5 [1.8–5.0]	3.0 [1.0–5.0]	Z = -1.786	.074
Volunteer patient sitter		1.0 [1.0–2.0]	2.0 [1.0–4.0]	Z = -1.225	.221
**Age** [years], *med* [IQR]		83.0 [74.0–88.0]	75.0 [64.3–84.0]	Z = -5.291	**< .001**
**Sex**, *n*(%)					
Female		132 (49.4)	130 (45.1)	χ^2^ = .862	.353
Male		135 (50.6)	158 (54.9)		
**Main medical diagnosis ICD-10 chapters**, *n*(%)		*n* = 263	*n* = 281		
Injury/poisoning of external cause		117 (44.5)	18 (6.4)	χ^2^ = 189.401	**< .001**
Neoplasms		42 (16.0)	57 (20.3)		
Diseases of the circulatory system		32 (12.2)	43 (15.3)		
Mental and behavioral diseases		2 (0.8)	42 (14.9)		
Diseases of the respiratory system		1 (0.4)	31 (11.0)		
Diseases of the musculoskeletal system		30 (11.4)	7 (2.5)		
Diseases of the nervous system		3 (1.1)	26 (9.3)		
Diseases of the genitourinary system		9 (3.4)	14 (5.0)		
Diseases of the digestive system		16 (6.0)	8 (2.8)		
Other ICD-10 chapters		11 (4.2)	35 (12.5)		
**Medical diagnosis**, *n*(%)					
Dementia		51 (19.1)	32 (11.1)	χ^2^ = 6.341	**.012**
Delirium		109 (40.8)	143 (49.7)	χ^2^ = 4.008	**.045**
Disorders due to use of alcohol		15 (5.6)	30 (10.4)	χ^2^ = 3.662	.056
Agitation & physical violence		0 (0.0)	4 (1.4)	-	.125
Suicidal ideation		3 (1.1)	4 (1.4)	-	>.999
**Behaviors and risk factors for patient sitter use**, *n/N* (%)					
Disorientation		193/222 (86.9)	206/231 (89.2)	χ^2^ = .349	.555
Inappropriate behavior		186/222 (83.8)	206/231 (89.2)	χ^2^ = 2.382	.123
Limited orientation		155/234 (66.2)	198/244 (81.1)	χ^2^ = 12.986	**< .001**
Risk of falls		209/235 (88.9)	222/247 (89.9)	χ^2^ = .035	.851
Intake of psychotropic drugs		63/235 (26.8)	83/247 (33.6)	χ^2^ = 2.321	.128
**In-hospital mortality**, *n/N* (%)		17/263 (6.5)	54/281 (19.2)	χ^2^ = 18.363	**< .001**

*Note*. *n* = number; *N* = sample size; *med* = median; IQR = interquartile range; *p* values in bold = statistically significant (*p* < .05)

### Organizational factors contributing to patient sitter use

**Fig 2:**
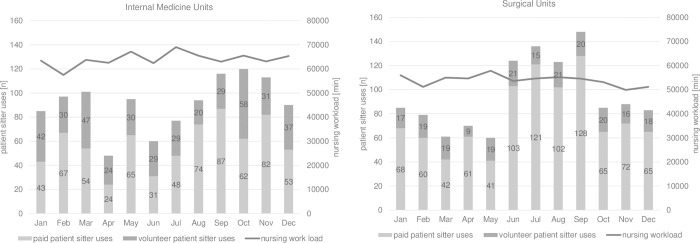
Nursing workload and patient sitter uses in internal medicine and surgical units. The bar chart shows the use of paid and volunteer patient sitters per month, from January to December 2018, in internal medicine (left panel) and surgical units (right panel). The line shows nursing workload in minutes per month.

Nursing workload measured in minutes was higher in internal medicine units than in surgical units, with a minimum of 57,515 minutes in February and a maximum of 69,065 minutes in July for internal medicine units (Fig 2). Nursing workload minutes in surgical units varied between 49,927 minutes in November and 57,854 minutes in May (Fig 2). In surgical units, the months with the highest number of patient sitter uses were September (n = 148), July (n = 136), June (n = 124) and August (n = 123) (Fig 2). In internal medicine units, patient sitter usage was highest in October (n = 120), September (n = 116), and November (n = 113), although variations throughout the year were more evenly distributed than in the surgical units (Fig 2). The total number of patient sitter uses was 1,144 for all surgical units and 1,096 for all internal medicine units. The distribution of paid and volunteer patient sitters varied considerably between the two departments. While 81.3% of surgical unit patient sitters were paid, only 63.0% were paid in internal medicine units. Two units (out of 20 in total) had more volunteer than paid patient sitter uses: the palliative care unit (70.2% volunteer) and a private internal medicine unit (60% volunteer). Considering all internal medicine and surgical units together, 27.7% (n = 620) of patient sitter uses were volunteer and 72.3% (n = 1,618) were paid.

## Discussion

This study aimed to enhance the body of knowledge on patient sitter use in acute care settings in the Swiss health care context. The results of this study provide evidence about the extent of the use of patient sitters in a Swiss acute care hospital, characteristics of patients requiring a patient sitter, the frequency of the use of patient sitters among different patient groups and contributing organizational factors.

The overall rate of patient sitters used in acute care patients is rarely stated in the existing literature. The findings of this study show a patient sitter use rate of 2.3%, which is comparable to the rate of 2.7% obtained in the Canadian study of Rochefort et al.’s [19] and the rate of 2.2% found by Thomann et al. [31] in Swiss and Austrian hospitals. The median sitter duration of 18 hours per patient in this study is, with less than a day, much shorter than in other studies. For example, Solimine et al. [1] found a mean duration of 1.4 to 3.8 days, depending on the age of the patients, Blumenfield et al. [32] one of 7.5 days; and in Al-Asmary et al. [33] the rate varied between 3.7 to 9.2 days. One explanation for this discrepancy might be that at the study hospital, the need for a patient sitter is evaluated separately for each shift, and patient sitters are rarely used for 24 hours. This contrasts with findings wherein most of the patient sitter requests (72%) were for 24-hour durations [34].

The median age of patients requiring a patient sitter was high in our study (78 years with three-quarters of patients being over 64 years old) compared to other studies, which found mean ages of 39 to 46 years [16, 32, 33]. The older age of patients with patient sitters may be reflected the high prevalence of delirium and dementia in this study’s population. These findings correspond with the results of Wood et al. [2], who found that dementia and delirium were the most common reasons for sitter usage. This is consistent with the evidence indicating that “injuries/poisoning of external cause” is a known risk factor for delirium, and accordingly, the most common principal medical diagnosis in patients with a delirium [35]. The high prevalence of dementia and delirium was even exceeded by the high rates of disorientation, inappropriate behavior, and risk of falls in the nursing assessments, suggesting that the need of a patient sitter is more dependent on behavior than on a diagnosis and that not every symptom of delirium or dementia is recognized or diagnosed as such.

Surprisingly low was the use of patient sitters in patients with suicidal ideation, especially compared to studies in which suicide risk was the most common reason for patient sitter usage [1, 13, 15, 16]. One reason for this might be that there is a nearby external psychiatric clinic, where patients with suicidal ideation or attempt would be initially admitted and to which the hospital makes referrals as soon as possible.

In-hospital mortality was higher in patients with a patient sitter than in patients without a patient sitter in this study. This may not only be corresponding with the high prevalence of delirium which is associated with higher mortality [36] but may also reflect severity of illness in general; the fact that patients with volunteer patient sitters had the highest mortality rate may indicate that volunteer patient sitters are more likely to be used in terminal care. This assumption is supported by the high percentage of volunteer patient sitters at the palliative care unit in this study, making volunteers an important segment of patient sitters in the study hospital. Other programs of which volunteers are an essential part have shown that they can lead to a significant reduction in rates of paid one-to-one care [37, 38], episodes of delirium [39], and overall costs [38]. Therefore, according to Carr [12], trained volunteers are the most cost-effective patient sitters. When it comes to the use of volunteer patient sitters in end-of-life care, Brighton et al. [40] emphasize that policymakers must not overlook the role of volunteers in hospitals including their training and support needs. Furthermore, the association between higher mortality and patient sitter use might indicate that the need for a patient sitter could be a predictor of poor patient outcome but the results provide only preliminary evidence that needs further research.

Our data show that internal medicine and surgical patients who required a patient sitter differed statistically significant by age, main medical diagnosis, prevalence of dementia and delirium, limited orientation, and in-hospital mortality. Patients requiring a patient sitter on surgical units were significantly older than those on internal medicine wards. Geriatric patients undergoing surgery are particularly at risk for poor outcomes, such as complications, mortality, and frailty, especially for emergency surgeries [41]. The older or frailer these surgical patients are, the more likely is their need for a patient sitter due to dementia, delirium, or risk of falls. This could also be the reason why surgical patients with patient sitters are more often patients with dementia, as a surgery may add mobilization restrictions or limitations or a general increase in morbidity and severity of illness for these already vulnerable patients. The statistically significant differences between the main medical diagnoses of surgical and internal medicine patients requiring a patient sitter are most likely to be reflected by the typical differences in diagnoses of internal medicine and surgical patients and therefore do not necessarily represent a difference in patient sitter use. This may also be the case for the differences in in-hospital mortality between the two groups. The focus on end-of-life and terminal care for patient sitters may be particularly important for internal medicine patients, considering that our data show that almost every fifth patient with a patient sitter died during hospitalization.

The figures on organizational factors did not clearly show that the amount of nursing workload minutes had an impact on patient sitter use; the figures did not show that higher workload was associated with a higher use of patient sitters. Although the figures should be interpreted with caution because they do not demonstrate a correlation between nursing workload and patient sitter use. Furthermore, overtime of nurses, as Rochefort et al. [19] investigated in their study, is not visible in the number for nursing workload in our study. We could, however, display the distribution of nursing workload and patient sitter uses over the course of a year. Our findings indicated that more patient sitters were required in the summer months in surgical units. On the other hand, in internal medicine units, patient sitter uses were more evenly distributed over the year but reached a peak in autumn. Finding reasons for the seasonality of patient sitter use is highly speculative (e.g., influenza season or heat waves in the summer of 2018 with inadequate fluid intake may have impacted the prevalence of delirium and therefore patient sitter use) and no evidence from existing literature could be found. Unfortunately, due to limited resources, we were not able to include nurse staffing characteristics in our calculations of organizational factors; such characteristics, especially understaffing and overtime, have previously been associated with higher patient sitter usage [19, 20].

Moreover, it is of widespread interest to reduce costs in patient sitter use [15, 19]. The Head of Nursing Development of the study hospital confirmed in discussions with the researchers that this was also the case for the study hospital. Nevertheless, we did not analyze patient sitter cost for similar reasons as Worley et al. [15], who noted that most hospitals lacked a tracking system for monitoring the costs and use of patient sitters. The findings of this study may be a good starting point for developing an algorithm for obtaining a patient sitter, as Salamon and Lennon [8] demonstrated that this leads to effective cost reduction. Others call for a transformation of the scope of responsibilities and activities for patient sitters [10, 42]. But before doing so, it is necessary to describe the scope of responsibilities and activities of patient sitters, as Moghabghab [11] did, and should be further researched.

This study has several strengths and limitations that should be acknowledged. One strength is that the study considered all eligible patients, hospitalized in the study hospital over the course of a full calendar year. Further, we examined the differences in the use of patient sitters between surgical and internal medicine patient, which has, to our knowledge, not been done before. The same may be true for the association between nursing workload and patient sitter use, as well as the distribution of patient sitter usage over the year. One limitation of this study is that in the involved hospital the use of patient sitters as well as the reason for the need of a patient sitter was not recorded directly. Because of this, the use of patient sitters had to be calculated based on the one-to-one nurse to patient ratio documented in the database LEP. Based on the discussion with the responsible persons in the hospital, this was the most appropriate way to identify the patients with a patient sitter. However, we cannot exclude the possibility that with this method, patients were included who had a one-to-one nurse patient ratio for other reasons, such as patients requiring a close monitoring because of a complex and invasive treatment with a high risk for complications. Further limitations of this study were the observational design and the inclusion of only one study site, both of which limit the generalizability of the results and causal statements. Other limitations may be the descriptive approach to the association between nursing workload and patient sitter use as well as the lack of comparison to patients without a patient sitter, which limits our ability to provide a holistic insight into patient sitter use, patient characteristics and the association of patients sitter use with organizational factors. However, this study design was the most feasible option for this study with respect to time, cost, and resources.

## Conclusions

In conclusion, this study makes an important contribution to the still-limited body of knowledge on patient sitter use, especially in acute care hospitals in Switzerland. Our findings that patient sitters were most frequently used for delirious and geriatric patients supports previous findings. The subgroup analysis and the shown use of patient sitter throughout the year need to be confirmed, preferably with a prospective study design, and should furthermore be pursued by persons responsible for nursing quality at the study hospital. Additionally, total cost analyses of the use of patient sitters not only including personnel costs but also saved costs of possible prevented adverse events, the impact and effectiveness of patient sitter use, patient and nurse experiences and needs (both, those of RNs and those of patient sitters) should be examined. Policymakers should consider developing patient sitter training and support programs, criteria for patient sitter qualification, as well as local policies for patient sitter use according to the patients’ needs. Under these circumstances and with the present findings, we trust that the call for clear (international) guidelines and policies on patient sitter use will eventually be answered.
