# Supplementary material for: The use of patient sitters at a Swiss hospital: A retrospective observational study
Source: PLoS One. 2023 Jun 14;18(6):e0287317. doi: 10.1371/journal.pone.0287317 (PMC10266662; doi:10.1371/journal.pone.0287317)
Supplement: Attachment — Submitted filename: Response to Reviewers.docx [file pone.0287317.s001.docx]

**Response to Reviewers**

| Academic Editor's / Reviewer's comment | Response |
| --- | --- |
| Academic Editor |  |
| Please ensure that your manuscript meets PLOS ONE's style requirements, including those for file naming. | Thank you very much for taking our manuscript into consideration for publication, your comments were very helpful to improve the quality of the manuscript.  Thank you for your reminder to check PLOS ONE's style requirements. We did so and made sure to meet the file naming requirements for the revised submission. Please let us know if we missed any other style requirements. |
| Please provide additional details regarding participant consent. In the ethics statement in the Methods and online submission information, please ensure that you have specified what type you obtained (for instance, written or verbal, and if verbal, how it was documented and witnessed). If your study included minors, state whether you obtained consent from parents or guardians. If the need for consent was waived by the ethics committee, please include this information. | We clarified the ethics statement in the Methods and materials section by explaining that the need for informed consent was not required according to national and institutional guidelines, because the Ethical Committee of the Canton of Zurich classified the project as not falling under the Swiss Human Research Act as only encrypted, routine patient data were used in this study.  Thank you for adapting the online submission information accordingly. |
| Thank you for stating the following financial disclosure:  “No third-party funding has been received for this study; the study was funded with own funds of the involved institutions (ZHAW Zurich University of Applied Sciences and Cantonal Hospital Winterthur).”  At this time, please address the following queries:  a) Please clarify the sources of funding (financial or material support) for your study. List the grants or organizations that supported your study, including funding received from your institution.  b) State what role the funders took in the study. If the funders had no role in your study, please state: “The funders had no role in study design, data collection and analysis, decision to publish, or preparation of the manuscript.”  c) If any authors received a salary from any of your funders, please state which authors and which funders.  d) If you did not receive any funding for this study, please state: “The authors received no specific funding for this work.”  Please include your amended statements within your cover letter; we will change the online submission form on your behalf. | Thank you for your questions for clarification of the financial disclosure.  After careful consideration we would like to change the statement to: “The authors received no specific funding for this work.”  We included the amended statement in our cover letter. Thank you for changing the online submission form on our behalf. |
| In your Data Availability statement, you have not specified where the minimal data set underlying the results described in your manuscript can be found. PLOS defines a study's minimal data set as the underlying data used to reach the conclusions drawn in the manuscript and any additional data required to replicate the reported study findings in their entirety. All PLOS journals require that the minimal data set be made fully available. For more information about our data policy, please see <http://journals.plos.org/plosone/s/data-availability>.  Upon re-submitting your revised manuscript, please upload your study’s minimal underlying data set as either Supporting Information files or to a stable, public repository and include the relevant URLs, DOIs, or accession numbers within your revised cover letter. For a list of acceptable repositories, please see <http://journals.plos.org/plosone/s/data-availability#loc-recommended-repositories>. Any potentially identifying patient information must be fully anonymized.  Important: If there are ethical or legal restrictions to sharing your data publicly, please explain these restrictions in detail. Please see our guidelines for more information on what we consider unacceptable restrictions to publicly sharing data: <http://journals.plos.org/plosone/s/data-availability#loc-unacceptable-data-access-restrictions>. Note that it is not acceptable for the authors to be the sole named individuals responsible for ensuring data access.  We will update your Data Availability statement to reflect the information you provide in your cover letter. | Unfortunately, there are ethical and legal restrictions to share the data publicly. Please update our Data Availability statement as follows: "Data cannot be shared publicly as they contain sensitive patient information. Data requests may be sent to Regina Krump, Head of Nursing Development, Kantonsspital Winterthur, 8401 Winterthur, Switzerland, regina.krump@ksw.ch."  We also included this statement in the cover letter. Thank you for updating our Data Availability statement. |
| Reviewer #1 |  |
| Abstract:  Line 26: “voluntary sitter”: familiar? Hospital service? Are they been paid or it is full voluntary? | Thank you very much for your comprehensive review, we appreciate it a lot.  Thank you for your question regarding the volunteer patient sitters. The volunteer patient sitters at this hospital do their service fully voluntary, they do not receive any payment and are not family members of the patients. After consideration, for easy readability, we would like to keep this expression in the abstract as it is and then explain the volunteer patient sitters in the manuscript text. Therefore, we added the information about payment and relationship with the patient in the chapter with the patient sitter descriptions (line 124 in the revised manuscript). We hope the concept of the volunteer patient sitters at the study hospital is now clearer and we hope you agree with this approach. |
| Abstract:  Line 26: “median duration” a day? Week? total stay? | Thank you for the question for clarification. The median duration stated was the duration per patient per stay. We added this information in the abstract (line 26 of the revised manuscript), as well as in the in the methods section in the sub chapter "study variables" (line 143) and in the results section in the first sub chapter "extent of patient sitter use" (lines 228 and 230). |
| Introduction:  Line 77: this paragraph solves a lot of doubts that arises me when I have started to read the manuscript. Could you try to place it before? | Thank you for your suggestion, which we glady implemented, it is now in the second paragraph of the introduction. We hope that this will make the introduction easier to follow. |
| Introduction:  Line 115: delete the “W” at the end of the paragraph. | Thank you for noticing this typing error. We deleted it. |
| Introduction must be rewritten. Content is quite good but I cannot find a logical order. Start with the definition and characteristics that these sitters have and after that explain that literature and different settings have in relation with this figure. | Thank you for your suggestion. We rewrote the introduction to simplify and clarify it. |
| Materials and methods:  Line 118 and 119: this sentences does not make sense. Rewrite. | Thank you. In order to make our statement about the study design clearer, we rewrote the first sentence of this paragraph and deleted the second sentence (lines 96-97 of the revised manuscript). |
| Materials and methods:  Line 119: “certain characteristics”: which ones? | This question has been resolved by the rewriting of the paragraph, see question above. |
| Materials and methods:  Line 125: sitters are part of the paid professionals who work in this hospital, isn´t it? | Thank you for your question. There are two types of patient sitter at the study hospital: paid and volunteer. The paid patient sitters are indeed part of the nursing workforce at the hospital, whereas the volunteer patient sitters are not professionals and not employed by the hospital but assigned by the chaplaincy team associated with the hospital. To clarify that this paragraph is about the hospital description and not yet about the patient sitters, we substituted the "they" in the last sentence with "these departments" (line 104 of the revised manuscript). |
| Materials and methods:  Line 133-154: I understand that there is no consensus about the characteristics of this figure must do (type of patient, type of care, number of hours…) but this section looks a bit abstract, because there is any line that justify a standard work of the sitter person. | Thank you for very much for your remark. We added a sentence to describe the patient sitters' responsibilities which should now complete the description of the patient sitters at the study hospital and make the picture less abstract (lines 131-134). |
| Materials and methods:  Line 177, data collection: if the sitter is a voluntary familiar, How did you collect data (number of hours for example)? | Thank you for your question. Your question made us realize that we forgot the crucial word "volunteer" when describing the data provided by the chaplaincy record. All data on volunteer patient sitters, such as the number of hours of one-to-one care for a patient they provided is documented in the chaplaincy record. We now added this important information (line 189). |
| Materials and methods:  Line 223: “the large amount of test” is not a justification for not to ask for an informed consent. Patients must be informed about their data will be analyzed. | Thank you for your note. We adjusted the information regarding informed consent according to national and institutional guidelines based on the editors' comment. |
| Results:  Line 232-233: some comment mention at the abstract section, daily median hours (of course not, it is an example for you), weekly?... | Thank you again for your comment. As previously noted, we adapted the measurement unit in the abstract, the methods, and results chapter. |
| Results:  Line 233: if you write numbers with numbers, please write 4 instead of “four”. | We followed the common rule of writing out numbers less than 10 in full to ensure readability. However, because in this particular sentence we reference a study result, we revised the sentence according to your suggestion (line 229). |
| Results:  Line 255, table 1: although men is the rest of the sample, please add this information in the table too. | Thank you, we added the information in the table. |
| Results:  Line 294: please try to improve the quality of both figures. | We uploaded all figures to the Preflight Analysis and Conversion Engine (PACE) to ensure that the figures meet the PLOS requirements. There were no image problems found, therefore we assumed the quality of the figures was sufficient. |
| Discussion:  Line 327: you do not mention nothing about suicidal in your results, or in general any specific cause for the need of a sitter. | The prevalence of suicidal ideation among patients requiring a patient sitter is stated in Table 1 in the results section. To prevent repetition, we did not mention the number again in the text of the results section.  Thank you very much though for noticing that we did not describe the specific reasons for the need of a patient sitter. Unfortunately, we could not include this a study variable as such because it was not documented at the study hospital. However, we included this information in the description of the study variables in the methods section to enhance transparency of our study variables (lines 144-148). We also added it as a limitation of our study (line 394). |
| Discussion:  Line 332: some comment regarding the mortality. | We are very sorry, we do not know exactly what you mean by your comment. Could you please rephrase your comment regarding the in-hospital mortality in the discussion section? |
| Additional comments:  Are there any criteria to assign this sitter to patients? | Thank you for your question. Unfortunately, there are not written standard criteria. However, there is an unofficial understanding regarding patient sitter assignment at the study hospital which we included in the subchapter "patient sitters in the study hospital" (lines 131-134). We hope the description of patient sitters is now clearer. |
| Additional comments:  I have no clear if this figure is part of the hospital or a familiar of the patient…. | We are sorry that this caused so much confusion. Thank you for your questions about this matter, we really hope it is clear now that the patient sitters (both paid and volunteer) are not family members of the patient. |
| Additional comments:  If patient is a familiar number of hours are often too much than personal care professional with a schedule (conditioning by the visit regime of the hospital), besides, if the sitter is a familiar, does not affect the hospital organization. | We agree, if the patient sitters were family of the patients, we would have had other research questions, probably more relevant to the families than the hospital. |
| Additional comments:  There is no mention about the cost of these sitters in your study. | Yes, this is true that we did not analyze the costs involved in patient sitter use. We did, however, discuss this in the last paragraph of the discussion section before the strengths and limitations (lines 378-387 of the revised manuscript). |
| Additional comments:  It is not clear us sitters are always nurses in your study or not, because you have not explained anything about this issue in the sitters mentioned in this sample. | We updated several sections and the chapter titles of the manuscript to hopefully make this issue clearer, as described previously. The study sample were the patients who required a patient sitter and not the patient sitters themselves. |
| Reviewer #2 |  |
| Introduction:  I think that the introduction should be simplified by using expressions that indicate the importance of the subject. | Thank you very much for your valuable review, we appreciate it a lot.  Thank you for your suggestion regarding the introduction. Based on your and Reviewer 1’s suggestion to rewrite the introduction, we did so and hope it is now simplified and the importance of the subject is clearer. |
| Introduction:  ‘’On the other hand, the kind of continuous observation that patient sitters provide is considered a restriction of personal liberty and thus, too, qualifies as a restraint measure under the Children and Adult Protection Act, which came into effect in Switzerland on January 1, 2013.’’ If the type of observation provided by patient sitters for these statements you discussed in the introduction is considered to be a restriction of freedom, why was it necessary to conduct such a study? Question must be answered. | Thank you for your interesting question. Continuous care for patients who are at a danger to themselves or others provided by patient sitters lies in a field of tension between autonomy and prevention of harm or between being illegal (for patients with full mental capacity) and medically necessary (for patients with limited mental capacity with the potential to harm themselves or others). It is therefore crucial to conduct a study to analyze this kind of care closely and raise awareness for this continuous observation and the patients it concerns.  In order to simplify and specify the whole introduction, we decided to delete this passage as the legal and ethical issues are not the focus of the study and should therefore not distract readers from the topic of the paper. |
| Materials and methods:  In the "Study sample" section, you should explain why patients who required at least four hours of paid or volunteer patient sitters were included in the study. | Thank you very much, we agree that it is necessary to explain the minimum of four hours of patient sitter use as our inclusion criteria. We added this information in the description of the study sample (lines 107-110 of the revised manuscript). |
| Materials and methods:  By making the definitions of volunteer and paid patient sitter broader, you should explain more clearly who is volunteer and who is paid. | Thank you for your comment. We tried to make the statements about paid and volunteer patient sitters clearer by adding "paid" or "volunteer" more often in the description of the patient sitters at the study hospital. |
| Materials and methods:  How is the usage period of the patient sitter determined in the system? Is there any clear information as you stated? | Thank you for your question. The hours of patient sitter usage for paid patient sitters were determined by the so called LEP database in which nurses document the time used for nursing activities related to patient care which includes "1:1-care". As for the volunteer patient sitters the hours they provided were stated in the chaplaincy record. We added these statements in the "data collection" subchapter (lines 183-186 of the revised manuscript). We also revised the description of the LEP database for more clarity (line 175 of the revised manuscript). |
| Materials and methods:  "Nursing workload measured in minutes" was this workload calculated based on what data? | The nursing workload was a variable automatically calculated by the LEP database. It was the sum of the time used for all nursing tasks documented by nurses per month. We added this information on line 165 in the revised manuscript. |
| Discussion:  The discussion part of the research needs to be elaborated a little more, for example, I think that the parts such as the months with the highest use of caregivers, the nursing workload should be discussed in the discussion or discussed in a more comprehensive way. | Thank you very much. We elaborated the discussion of the nursing workload associated with patient sitter use more (lines 362-367). We would have also liked to elaborate the discussion part of the seasonality of patient sitter uses, or rather the use of patient sitters over the course of a year a bit more by comparing it to existing evidence. Unfortunately, we could not find any literature regarding this topic. Therefore, the discussion stays based on speculation. But we added the information about the non-existence of the literature (line 374). |
