# Supplementary material for: The use of patient sitters at a Swiss hospital: A retrospective observational study
Source: PLoS One. 2023 Jun 14;18(6):e0287317. doi: 10.1371/journal.pone.0287317 (PMC10266662; doi:10.1371/journal.pone.0287317)
Supplement: Peer review history

# Peer review: Decision Letter 0

**DOI:** 10.1371/journal.pone.0287317.r001

**Authors:** Bora Çekmen

2 Jan 2023

PONE-D-22-29007The use of patient sitters at a Swiss hospital: A retrospective observational studyPLOS ONE

Dear Dr. Kramer,

Thank you for submitting your manuscript to PLOS ONE. After careful consideration, we feel that it has merit but does not fully meet PLOS ONE’s publication criteria as it currently stands. Therefore, we invite you to submit a revised version of the manuscript that addresses the points raised during the review process.

Your article was considered interesting. Many revisions are required. Reviewer opinions also support this issue. If you make these corrections completely, it will be evaluated by us again. Please fully comply with all reviewer comments.

Please submit your revised manuscript by Feb 16 2023 11:59PM. If you will need more time than this to complete your revisions, please reply to this message or contact the journal office at plosone@plos.org. When you're ready to submit your revision, log on to https://www.editorialmanager.com/pone/ and select the 'Submissions Needing Revision' folder to locate your manuscript file.

Please include the following items when submitting your revised manuscript:

- A rebuttal letter that responds to each point raised by the academic editor and reviewer(s). You should upload this letter as a separate file labeled 'Response to Reviewers'.
- A marked-up copy of your manuscript that highlights changes made to the original version. You should upload this as a separate file labeled 'Revised Manuscript with Track Changes'.
- An unmarked version of your revised paper without tracked changes. You should upload this as a separate file labeled 'Manuscript'.

If you would like to make changes to your financial disclosure, please include your updated statement in your cover letter. Guidelines for resubmitting your figure files are available below the reviewer comments at the end of this letter.

If applicable, we recommend that you deposit your laboratory protocols in protocols.io to enhance the reproducibility of your results. Protocols.io assigns your protocol its own identifier (DOI) so that it can be cited independently in the future. For instructions see: https://journals.plos.org/plosone/s/submission-guidelines#loc-laboratory-protocols. Additionally, PLOS ONE offers an option for publishing peer-reviewed Lab Protocol articles, which describe protocols hosted on protocols.io. Read more information on sharing protocols at https://plos.org/protocols?utm_medium=editorial-email&utm_source=authorletters&utm_campaign=protocols.

We look forward to receiving your revised manuscript.

Kind regards,

Bora Çekmen

Academic Editor

PLOS ONE

Journal Requirements:

When submitting your revision, we need you to address these additional requirements.

1. Please ensure that your manuscript meets PLOS ONE's style requirements, including those for file naming. The PLOS ONE style templates can be found at

https://journals.plos.org/plosone/s/file?id=wjVg/PLOSOne_formatting_sample_main_body.pdf and

https://journals.plos.org/plosone/s/file?id=ba62/PLOSOne_formatting_sample_title_authors_affiliations.pdf

2. Please provide additional details regarding participant consent. In the ethics statement in the Methods and online submission information, please ensure that you have specified what type you obtained (for instance, written or verbal, and if verbal, how it was documented and witnessed). If your study included minors, state whether you obtained consent from parents or guardians. If the need for consent was waived by the ethics committee, please include this information.

3. Thank you for stating the following financial disclosure:

“No third-party funding has been received for this study; the study was funded with own funds of the involved institutions (ZHAW Zurich University of Applied Sciences and Cantonal Hospital Winterthur).”

At this time, please address the following queries:

a)        Please clarify the sources of funding (financial or material support) for your study. List the grants or organizations that supported your study, including funding received from your institution.

b)        State what role the funders took in the study. If the funders had no role in your study, please state: “The funders had no role in study design, data collection and analysis, decision to publish, or preparation of the manuscript.”

c)        If any authors received a salary from any of your funders, please state which authors and which funders.

d)        If you did not receive any funding for this study, please state: “The authors received no specific funding for this work.”

Please include your amended statements within your cover letter; we will change the online submission form on your behalf.

4. In your Data Availability statement, you have not specified where the minimal data set underlying the results described in your manuscript can be found. PLOS defines a study's minimal data set as the underlying data used to reach the conclusions drawn in the manuscript and any additional data required to replicate the reported study findings in their entirety. All PLOS journals require that the minimal data set be made fully available. For more information about our data policy, please see http://journals.plos.org/plosone/s/data-availability.

Upon re-submitting your revised manuscript, please upload your study’s minimal underlying data set as either Supporting Information files or to a stable, public repository and include the relevant URLs, DOIs, or accession numbers within your revised cover letter. For a list of acceptable repositories, please see http://journals.plos.org/plosone/s/data-availability#loc-recommended-repositories. Any potentially identifying patient information must be fully anonymized.

Important: If there are ethical or legal restrictions to sharing your data publicly, please explain these restrictions in detail. Please see our guidelines for more information on what we consider unacceptable restrictions to publicly sharing data: http://journals.plos.org/plosone/s/data-availability#loc-unacceptable-data-access-restrictions. Note that it is not acceptable for the authors to be the sole named individuals responsible for ensuring data access.

We will update your Data Availability statement to reflect the information you provide in your cover letter.

[Note: HTML markup is below. Please do not edit.]

Reviewers' comments:

Reviewer's Responses to Questions

**Comments to the Author**

1. Is the manuscript technically sound, and do the data support the conclusions?

The manuscript must describe a technically sound piece of scientific research with data that supports the conclusions. Experiments must have been conducted rigorously, with appropriate controls, replication, and sample sizes. The conclusions must be drawn appropriately based on the data presented. 

Reviewer #1: Partly

Reviewer #2: Partly

**********

2. Has the statistical analysis been performed appropriately and rigorously? 

Reviewer #1: I Don't Know

Reviewer #2: Yes

**********

3. Have the authors made all data underlying the findings in their manuscript fully available?

The PLOS Data policy requires authors to make all data underlying the findings described in their manuscript fully available without restriction, with rare exception (please refer to the Data Availability Statement in the manuscript PDF file). The data should be provided as part of the manuscript or its supporting information, or deposited to a public repository. For example, in addition to summary statistics, the data points behind means, medians and variance measures should be available. If there are restrictions on publicly sharing data—e.g. participant privacy or use of data from a third party—those must be specified.

Reviewer #1: Yes

Reviewer #2: Yes

**********

4. Is the manuscript presented in an intelligible fashion and written in standard English?

PLOS ONE does not copyedit accepted manuscripts, so the language in submitted articles must be clear, correct, and unambiguous. Any typographical or grammatical errors should be corrected at revision, so please note any specific errors here.

Reviewer #1: Yes

Reviewer #2: Yes

**********

5. Review Comments to the Author

Please use the space provided to explain your answers to the questions above. You may also include additional comments for the author, including concerns about dual publication, research ethics, or publication ethics. (Please upload your review as an attachment if it exceeds 20,000 characters)

Reviewer #1: Manuscript shows the inpatients sitter figure during one year.

Abstract.

Line 26: “voluntary sitter”: familiar? Hospital service? Are they been paid or it is full voluntary?

Line 26: “median duration” a day? Week? total stay?

Introduction.

Line 77: this paragraph solves a lot of doubts that arises me when I have started to read the manuscript. Could you try to place it before?

Line 115: delete the “W” at the end of the paragraph.

Introduction must be rewritten. Content is quite good but I cannot find a logical order. Start with the definition and characteristics that these sitters have and after that explain that literature and different settings have in relation with this figure.

Material and methods

Line 118 and 119: this sentences does not make sense. Rewrite.

Line 119: “certain characteristics”: which ones?

Line 125: sitters are part of the paid professionals who work in this hospital, isn´t it?

Line 133-154: I understand that there is no consensus about the characteristics of this figure must do (type of patient, type of care, number of hours…) but this section looks a bit abstract, because there is any line that justify a standard work of the sitter person.

Line 177, data collection: if the sitter is a voluntary familiar, How did you collect data (number of hours for example)?

Line 223: “the large amount of test” is not a justification for not to ask for an informed consent. Patients must be informed about their data will be analyzed.

Results.

Line 232-233: some comment mention at the abstract section, daily median hours (of course not, it is an example for you), weekly?...

Line 233: if you write numbers with numbers, please write 4 instead of “four”.

Line 255, table 1: although men is the rest of the sample, please add this information in the table too.

Line 294: please try to improve the quality of both figures.

Discussion.

Line 327: you do not mention nothing about suicidal in your results, or in general any specific cause for the need of a sitter.

Line 332: some comment regarding the mortality.

Additional comments:

Are there any criteria to assign this sitter to patients?

I have no clear if this figure is part of the hospital or a familiar of the patient….

If patient is a familiar number of hours are often too much than personal care professional with a schedule (conditioning by the visit regime of the hospital), besides, if the sitter is a familiar, does not affect the hospital organization.

There is no mention about the cost of these sitters in your study.

It is not clear us sitters are always nurses in your study or not, because you have not explained anything about this issue in the sitters mentioned in this sample.

Reviewer #2: Dear authors,

I think that the introduction should be simplified by using expressions that indicate the importance of the subject.

‘’On the other hand, the kind of continuous observation that patient sitters provide is considered a restriction of personal liberty and thus, too, qualifies as a restraint measure under the Children and Adult Protection Act, which came into effect in Switzerland on January 1, 2013.’’ If the type of observation provided by patient sitters for these statements you discussed in the introduction is considered to be a restriction of freedom, why was it necessary to conduct such a study? Question must be answered.

In the "Study sample" section, you should explain why patients who required at least four hours of paid or volunteer patient sitters were included in the study.

By making the definitions of volunteer and paid patient sitter broader, you should explain more clearly who is volunteer and who is paid. How is the usage period of the patient sitter determined in the system? Is there any clear information as you stated?

"Nursing workload measured in minutes" was this workload calculated based on what data?

The discussion part of the research needs to be elaborated a little more, for example, I think that the parts such as the months with the highest use of caregivers, the nursing workload should be discussed in the discussion or discussed in a more comprehensive way.

**********

6. PLOS authors have the option to publish the peer review history of their article (what does this mean?). If published, this will include your full peer review and any attached files.

If you choose “no”, your identity will remain anonymous but your review may still be made public.

**Do you want your identity to be public for this peer review?** For information about this choice, including consent withdrawal, please see our Privacy Policy.

Reviewer #1: No

Reviewer #2: No

**********

[NOTE: If reviewer comments were submitted as an attachment file, they will be attached to this email and accessible via the submission site. Please log into your account, locate the manuscript record, and check for the action link "View Attachments". If this link does not appear, there are no attachment files.]

While revising your submission, please upload your figure files to the Preflight Analysis and Conversion Engine (PACE) digital diagnostic tool, https://pacev2.apexcovantage.com/. PACE helps ensure that figures meet PLOS requirements. To use PACE, you must first register as a user. Registration is free. Then, login and navigate to the UPLOAD tab, where you will find detailed instructions on how to use the tool. If you encounter any issues or have any questions when using PACE, please email PLOS at figures@plos.org. Please note that Supporting Information files do not need this step.

# Author comment: Author response to Decision Letter 0

**DOI:** 10.1371/journal.pone.0287317.r002

15 Mar 2023

Dear Reviewers, thank you very much for your valuable review. Thanks to your reviews and comments, the manuscript has gained significantly in clarity and comprehensibility. Please find all answers to your comments in the document "Response to Reviewers." Thank you very much for the opportunity to revise our manuscript and for your re-evaluation to publish our manuscript.

Attachment Submitted filename: Response to Reviewers.docx Click here for additional data file.

# Peer review: Decision Letter 1

**DOI:** 10.1371/journal.pone.0287317.r003

**Authors:** Federica Canzan

5 Jun 2023

The use of patient sitters at a Swiss hospital: A retrospective observational study

PONE-D-22-29007R1

Dear Dr. Kramer,

We’re pleased to inform you that your manuscript has been judged scientifically suitable for publication and will be formally accepted for publication once it meets all outstanding technical requirements.

Within one week, you’ll receive an e-mail detailing the required amendments. When these have been addressed, you’ll receive a formal acceptance letter and your manuscript will be scheduled for publication.

An invoice for payment will follow shortly after the formal acceptance. To ensure an efficient process, please log into Editorial Manager at http://www.editorialmanager.com/pone/, click the 'Update My Information' link at the top of the page, and double check that your user information is up-to-date. If you have any billing related questions, please contact our Author Billing department directly at authorbilling@plos.org.

If your institution or institutions have a press office, please notify them about your upcoming paper to help maximize its impact. If they’ll be preparing press materials, please inform our press team as soon as possible -- no later than 48 hours after receiving the formal acceptance. Your manuscript will remain under strict press embargo until 2 pm Eastern Time on the date of publication. For more information, please contact onepress@plos.org.

Kind regards,

Federica Canzan

Academic Editor

PLOS ONE

Additional Editor Comments (optional):

Reviewers' comments:

Reviewer's Responses to Questions

**Comments to the Author**

1. If the authors have adequately addressed your comments raised in a previous round of review and you feel that this manuscript is now acceptable for publication, you may indicate that here to bypass the “Comments to the Author” section, enter your conflict of interest statement in the “Confidential to Editor” section, and submit your "Accept" recommendation.

Reviewer #1: All comments have been addressed

**********

2. Is the manuscript technically sound, and do the data support the conclusions?

The manuscript must describe a technically sound piece of scientific research with data that supports the conclusions. Experiments must have been conducted rigorously, with appropriate controls, replication, and sample sizes. The conclusions must be drawn appropriately based on the data presented. 

Reviewer #1: Yes

**********

3. Has the statistical analysis been performed appropriately and rigorously? 

Reviewer #1: Yes

**********

4. Have the authors made all data underlying the findings in their manuscript fully available?

The PLOS Data policy requires authors to make all data underlying the findings described in their manuscript fully available without restriction, with rare exception (please refer to the Data Availability Statement in the manuscript PDF file). The data should be provided as part of the manuscript or its supporting information, or deposited to a public repository. For example, in addition to summary statistics, the data points behind means, medians and variance measures should be available. If there are restrictions on publicly sharing data—e.g. participant privacy or use of data from a third party—those must be specified.

Reviewer #1: Yes

**********

5. Is the manuscript presented in an intelligible fashion and written in standard English?

PLOS ONE does not copyedit accepted manuscripts, so the language in submitted articles must be clear, correct, and unambiguous. Any typographical or grammatical errors should be corrected at revision, so please note any specific errors here.

Reviewer #1: Yes

**********

6. Review Comments to the Author

Please use the space provided to explain your answers to the questions above. You may also include additional comments for the author, including concerns about dual publication, research ethics, or publication ethics. (Please upload your review as an attachment if it exceeds 20,000 characters)

Reviewer #1: Dear Authors,

In relation with my comment (line 332), there is a mistake. I wanted to write "sAme comment regarding the mortality" (like my previous comment about suicidal information). Because before the discussion section any comment about mortality had been done.

Thank you very much for considering my suggestions. Manuscript looks much clerarer for me.

**********

7. PLOS authors have the option to publish the peer review history of their article (what does this mean?). If published, this will include your full peer review and any attached files.

If you choose “no”, your identity will remain anonymous but your review may still be made public.

**Do you want your identity to be public for this peer review?** For information about this choice, including consent withdrawal, please see our Privacy Policy.

Reviewer #1: No

**********

# Editor report: Acceptance letter

**DOI:** 10.1371/journal.pone.0287317.r004

**Authors:** Federica Canzan

6 Jun 2023

PONE-D-22-29007R1 

The use of patient sitters at a Swiss hospital: A retrospective observational study 

Dear Dr. Schubert:

I'm pleased to inform you that your manuscript has been deemed suitable for publication in PLOS ONE. Congratulations! Your manuscript is now with our production department. 

If your institution or institutions have a press office, please let them know about your upcoming paper now to help maximize its impact. If they'll be preparing press materials, please inform our press team within the next 48 hours. Your manuscript will remain under strict press embargo until 2 pm Eastern Time on the date of publication. For more information please contact onepress@plos.org.

If we can help with anything else, please email us at plosone@plos.org. 

Thank you for submitting your work to PLOS ONE and supporting open access. 

Kind regards, 

PLOS ONE Editorial Office Staff

on behalf of

Professor Federica Canzan 

Academic Editor

PLOS ONE
